# Genetic Background of Antimicrobial Resistance in Multiantimicrobial-Resistant *Escherichia coli* Isolates from Feces of Healthy Broiler Chickens in Tunisia

**DOI:** 10.1155/2021/1269849

**Published:** 2021-10-01

**Authors:** Mohamed Salah Abbassi, Hajer Kilani, Islem Abid, Yolanda Sáenz, Paul Hynds, Sana Lengliz, Noureddine Ben Chehida, Ilhem Boutiba-Ben Boubaker

**Affiliations:** ^1^Université de Tunis El Manar, Institut de la Recherche Vétérinaire de Tunisie, 20 Rue Jebel Lakhdhar, Bab Saadoun, Tunis 1006, Tunisia; ^2^Université de Tunis El Manar, Faculté de Médecine de Tunis, Laboratoire de Résistance Aux Antibiotiques LR99ES09, Tunisia; ^3^Department of Botany and Microbiology, College of Science, King Saud University, Riyadh 11451, Saudi Arabia; ^4^Área de Microbiología Molecular, Centro de Investigación Biomédica de La Rioja (CIBIR), Logroño, Spain; ^5^Environmental Sustainability and Health Institute (ESHI), Technological University Dublin, Grangegorman, Dublin 7, Dublin, Ireland

## Abstract

Multiantimicrobial-resistant *Escherichia coli* isolates are a global human health problem causing increasing morbidity and mortality. Genes encoding antimicrobial resistance are mainly harbored on mobile genetic elements (MGEs) such as transposons and plasmids as well as integrons, which enhance their rapid spread. The aim of this study was to characterize 83 multiantimicrobial-resistant *E. coli* isolates recovered from healthy broiler chickens. Among 78 tetracycline-resistant isolates, the *tetA*, *tetB*, and *tetC* genes were detected in 59 (75.6%), 14 (17.9%), and one (1.2%) isolates, respectively. The *sul1*, *sul2*, and *sul3* genes were detected 31 (46.2%), 16 (23.8%), and 6 (8.9%) isolates, respectively, among 67 sulfonamide-resistant isolates. The PCR-based replicon typing method showed plasmids in 29 isolates, IncFIB (19), IncI1-I*γ* (17), IncF (14), IncK (14), IncFIC (10), IncP (8), IncY (3), IncHI2 (1), and IncX (1). The class 1 and 2 integrons were detected in 57 and 2 isolates, respectively; one isolate harbored both integrons. Seven and one gene cassette arrays were identified in class 1 and class 2 integrons, respectively. Our findings show that multiantimicrobial-resistant *E. coli* isolates from chickens serve as reservoirs of highly diverse and abundant *tet* and *sul* genes and plasmid replicons. Such isolates and MGEs pose a potential health threat to the public and animal farming.

## 1. Introduction

*Escherichia coli* is a normal part of the microbiota of the lower gastrointestinal tract of warm-blooded animals and humans and usually exists as a harmless commensal. However, there also exist many pathogenic strains of *E. coli* that can cause a variety of diarrheal and other extraintestinal infections in humans and animals. The emergence of *E. coli* isolates with multiple antibiotic resistance phenotypes has been previously reported and is considered a serious health concern [1, 2]. In *Enterobacteriaceae* and particularly in *E. coli*, resistance to beta-lactams due to extended spectrum beta-lactamases (ESBL), quinolones, and aminoglycosides has drawn considerable attention worldwide [3]. ESBL-producing isolates are usually resistant to other antimicrobial agents such as aminoglycosides, tetracyclines, chloramphenicol, trimethoprim, sulfonamides, or quinolones, often due to the presence of multiple resistance genes on transferable genetic elements such as plasmids, transposons, or integrons [4–8]. In the last decade, it has been observed that ESBL producers and multiantimicrobial-resistant *E. coli* isolates are frequently detected in food-producing animals or food products; therefore, health authorities are worried about the potential transmission of these resistant microorganisms to humans through the food chain [2, 3, 9]. Likewise, in Tunisia, many reports have highlighted high rates of antimicrobial resistance and ESBL production in *E. coli* isolates from food-producing animals or food products [9–12], a situation that requires more investigations and vigilance to reduce large dissemination of such resistant isolates.

Mobile genetic elements such as plasmids, transposons, and integrons are able to disseminate genes encoding antibiotic resistance by horizontal transfer and play an important role in the evolution and dissemination of multiantimicrobial resistance in Gram-negative bacteria [13]. Five classes of integrons related to antimicrobial resistance have been described based on the homology of their integrase genes [14]. However, class 1 and 2 were the most prevalent integrons in *Enterobacteriaceae*. Class 1 integron comprises two conserved segments, the 5′ conserved sequence (5′CS) (bearing *int1* gene) and the 3′ conserved sequence (3′CS) (containing *qacEΔ-sul1* genes) and an internal variable region [5, 13, 14]. Variable regions are able to contain many gene cassettes, which might explain, in part, the multiantimicrobial resistance trait of some reported isolates [13, 14].

In Tunisia, little is known about the epidemiology of antimicrobial resistance in *Enterobacteriaceae*, especially in *E. coli*, of animal origin. However, recent studies highlighted high rates of antimicrobial-resistant *E. coli* isolates from avian farms [9–11]. Therefore, in this work, we aimed to study 83 multiantimicrobial-resistant *E. coli* isolates recovered from healthy broiler chickens by determining genes encoding tetracycline and sulfonamide resistance and the occurrence of integrons and plasmid types. This study will allow us to better understand the genetic background of antimicrobial resistance in these isolates.

## 2. Materials and Methods

### 2.1. Bacterial Strains and Epidemiological Background

A program of regular surveillance of antimicrobial resistance in zoonotic bacteria of animal origin has been established at 2009 by the laboratory of bacteriological research in the Tunisian Institute of Veterinary Research, Tunisia. According to this program, samples (feces, milk, meat, and organs) from various animals (healthy or sick) have been collected and analyzed to study antimicrobial susceptibilities of pathogenic strains or indicator species such as *Enterococcus* spp. and *E. coli*. Between June 2009 and December 2015, one hundred seventy fresh feces samples of healthy broiler chickens were collected from 8 unrelated intensive farms (each with 2000-5000 animals) in Siliana (*n* = 20), El-Kef (*n* = 20), Sidi Bouzid (*n* = 20), Beja (20), Nabeul (25), Bousalem (*n* = 20), Mateur (*n* = 20), and Sousse (*n* = 25) regions, Tunisia. The mean average age of the broilers was 38 days. The farms were selected by considering their geographical location and the size of the farms (at least 2000 chickens per farm). A single fecal dropping from each chicken was collected with sterile swab. Following sample collection, the samples were transported immediately to the laboratory in an insulating foam box with ice and were analyzed within 24 h. Five grams of feces was incubated in 5 mL of Brain Heart Infusion (BHI, Becton Dickinson) for 1 h at 37°C. After serial dilutions, 100 *μ*L of the suspension was streaked onto MacConkey Agar (MSA, Becton Dickinson) and incubated at 37°C for 18-24 h. One colony with typical *E. coli* trait from each sample was picked and reisolated on MacConkey's agar. Isolates were preidentified by Gram staining and classical biochemical (tests of oxidase, urease, indole, and growth on simmonse citrate) and then confirmed by biochemical identification using Api20E (Bio-Mérieux, France).

#### 2.2. Antimicrobial Susceptibility Testing

Antimicrobial susceptibility testing was carried out by the agar disk diffusion method on Mueller–Hinton agar plates according to therecommendation of *Clinical and Laboratory Standards Institute* guidelines [15]. The tested antimicrobial agents were amoxicillin (25 *μ*g), ticarcillin (75 *μ*g), imipenem (10 *μ*g), nalidixic acid (30 *μ*g), ciprofloxacin (5 *μ*g), sulfonamide (300 *μ*g), trimethoprim/sulfamethoxazole (1.25/23.75 *μ*g), tetracycline (30 *μ*g), gentamicin (10 *μ*g), kanamycin (30), streptomycin (10 *μ*g), and chloramphenicol (30 *μ*g). For all isolates, the double-disk synergy test (DDST) with cefotaxime or ceftazidime in the proximity to amoxicillin-clavulanic acid was used for the screening of ESBL [15]. *E. coli* ATCC25922 was used as ESBL negative, and *Klebsiella pneumoniae* 700603 was used as a ESBL-positive reference strain. Isolates demonstrating intermediate susceptibility or resistance to three or more classes of antimicrobial agents were classified as multidrug-resistant (MDR) [16] and were further characterized in this study.

### 2.3. Genomic DNA Extraction

Genomic DNA was extracted from each isolate using the boiling method. Briefly, a loopful of cell bacteria from an overnight culture on Mueller-Hinton Agar (Bio-Rad) was suspended in Eppendorf tube containing 500 *μ*L of DNase-RNase-free distilled water. The Eppendorf tube was incubated for 15 min at 100°C and placed immediately at -20°C for 10 min. The tube was centrifuged for 5 min at 10,000 × g at 4°C. The supernatant containing genomic DNA was stored at -20°C until use for PCR experiments.

### 2.4. Plasmid Typing

Plasmids were typed according to their incompatibility group (18 Inc groups) using the PCR-based replicon typing method [17].

### 2.5. Detection and Characterization of Class 1 and 2 Integrons

Class 1 and 2 integrons and the 3′ conserved sequence (3′CS) (*qacEΔ*-*sul1*) of class 1 integrons were amplified by PCR as previously reported [18]. In addition, the variable regions (VR) of class 1 and 2 the integrons were amplified and sequenced in 20 randomly selected class 1-positive isolates (one of them contained also class 2) and one class 2-positive isolate [18]. Amplicons of variable regions were purified through K501 spin columns Biomatik (Wilmington, DE) and were sequenced using appropriate primers, the AmpliTaq DNA polymerase FS Dye Terminator Cycle Sequencing Ready Reaction kit (Applied Biosystems, Courtaboeuf, France) and the automatic ABI Prism_3100 genetic analyzer (Applied Biosystems). The sequences were confirmed with those in the GenBank nucleotide database using the basic local alignment search tool (BLAST) program available through the National Center for Biotechnology Information website (http://www.ncbi.nlm.nih.gov/BLAST). For nonclassical class 1 integrons, VR were determined by the primer walking strategy [18].

### 2.6. Detection of the Genes Encoding Tetracycline and Sulfonamide Resistance and ESBL Production

The presence of genes encoding tetracycline (*tetA*, *tetB*, and *tetC*) and sulfonamide resistance (*sul1*, *sul2*, and *sul3*) was investigated by PCR for all resistant isolates to these markers as reported previously [18]. The presence of *bla*_TEM_, *bla*_SHV_, and *bla*_CTX-M_ genes associated to the production of ESBL was investigated by PCR with specific primers as previously described [12]. *bla* amplicons were purified and sequenced on both strands using appropriate primers as mentioned above. The nucleotide and their deduced amino acid sequences were compared with those included in the GenBank database using the BLAST tool of the National Center for Biotechnology Information website (http://www.ncbi.nlm.nih.gov/BLAST).

### 2.7. Statistical Analysis

Statistical analysis was performed by means of Pearson's chi-square test (with Yate's correction) to check if there were differences among integron-positive and integron-negative isolates in relation with antimicrobial resistance phenotypes and presence of *sul* and *tet* gene types.

## 3. Results

### 3.1. Susceptibility to Antibiotics

All the 170 fecal samples contained typical colonies of *E. coli*. One colony per sample was randomly selected, confirmed as *E. coli* par Api20E and tested for its susceptibility to antimicrobial agents. According to their antimicrobial susceptibility, among the 170 *E. coli* isolates, 83 (48.8%) were classified as multiantimicrobial resistant and were further characterized in this study. Among the 83 isolates, high rates of resistance were observed for tetracycline (78 isolates, 93.9%), sulfonamides (67 isolates, 80.7%), streptomycin (62 isolates, 74.6%), amoxicillin (55 isolates, 66.2%), nalidixic acid (46 isolates, 55.4%), and amoxicillin/clavulanic acid (36 isolates, 43.3%). Moderate frequencies of antimicrobial resistance were noted for ciprofloxacin and ticarcillin (each 27 isolates, 32.5%). Low frequencies were observed for kanamycin (7 isolates, 8.4%), cefotaxime (3 isolates, 3.6%), and ceftazidime (2 isolates, 2.4%). Interestingly only one ESBL-producing *E. coli* isolate was detected. Resistance to imipenem, gentamicin, and chloramphenicol were not detected.

### 3.2. Genes Encoding Tetracycline and Sulfonamide Resistance and ESBL Production

The *tetA*, *tetB*, and *tetC* genes were observed in 59 (75.6%), 14 (17.9%), and one (1.2%) tetracycline-resistant isolates, respectively. *tetA* and *tetB* genes were simultaneously found in 6 isolates. Sixty-seven isolates were sulfonamide resistant, and *sul*-type genes were detected in 44 (67.6%) of them. The *sul1*, *sul2*, and *sul3* genes were detected in 31 (46.2%), 16 (23.8%), and 6 (8.9%) resistant isolates, respectively. The combinations of following genes were identified (number of isolates): *sul1*+*sul2* (4), *sul1*+*sul3* (3), *sul2*+*sul3* (1), and *sul1*+*sul2*+*sul3* (1). It is interesting to note that isolates containing integrons were more resistant to different antimicrobial families than integron-free isolates (Tables 1 and 2). As shown in Table 2, 51.7% (*n* = 30) of integron-positive isolates showed resistance to at least five different antimicrobial families versus 24% (*n* = 6) of integron-negative isolates (statistically significant, *p* < 0.001).

It is also worth noting that we showed a positive correlation between the presence of integrons and the detection of genes encoding resistance to tetracycline and to sulfonamides (Table 3). Indeed out of 44 isolates harboring *sul*-type genes, 37 (84%) were integron positive (*p* < 0.001). Similarly, among 68 *tet* gene-positive isolates, 46 (67.6%) were integron positive (*p* < 0.001). The ESBL producer isolate was class 1 integron positive and contained the *bla*_CTX-M-1_, *tet*B, and *sul*1 genes.

### 3.3. Plasmid Incompatibility Groups

PCR was used to identify incompatibility plasmid groups (or plasmid replicon types) by using genomic DNA of the 83 multiantimicrobial-resistant *E. coli* isolates. Twenty-nine (34.9%) isolates carried at least one incompatibility plasmid group, including (number of isolates) IncFIB (19), IncI1-I*γ* (17), IncF (14), K (14), IncFIC (10), IncP (8), Y (3), IncHI2 (1), and IncX (1). Interestingly, 22 (26.5%) isolates harbored at least two plasmid replicon types and various combinations were detected (number of isolates): FIB+F+I1-I*γ* (3), FIB+F+K+Y (2), F+K+I1-I*γ* (2), FIB+FIC+K+I1-I*γ* (1), FIB+F+K+P (1), FIB+F+FIC+K+P (1), FIB+K+P (1), FIB+K+P+HI2 (1), FIC+K+Y (1), FIB+K+Y+I1-I*γ* (1), FIB+F+K+I1-I*γ* (1), FIB+F+P+I1-I*γ* (1), I1-I*γ*+FIB (1), FIB+F+X+I1-I*γ* (1), FIB+F+FIC+Y+I1-I*γ* (1), FIB+F+FIC+K (1), FIB+F+FIC+I1-I*γ*+K (1), and FIB+K (1).

### 3.4. Occurrence of Integrons and Variable Regions

**Figure 1:**
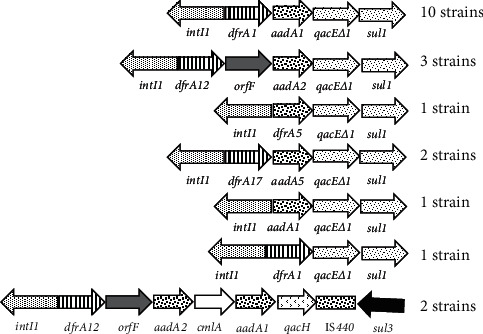
Gene cassette arrangements included in the class 1 integrons detected in 20 class 1 integron-positive *E. coli* strains. *IntI1*: gene encoding integrase of class 1 integron; *dfr1*, *dfrA5*, *dfrA12*, and *dfrA17*: genes encoding trimethoprim resistance; *aadA1* and *aadA2* and *aadA5*: genes encoding streptomycin/spectinomycin resistance; *qacE∆1*: gene encoding quaternary ammonium; *sul1* and *sul3* (in inversion direction): genes encoding sulfonamide resistance; *cmlA:* gene encoding chloramphenicol resistance. *orfF*: open reading frame of unknown function; IS*440:* insertion section 440.

Class 1 and 2 integrons were detected in 58 isolates. Fifty-seven isolates harbored the class 1 integrons, while class 2 was found only in two isolates. One isolate harbored both integrons. The 3′CS, mainly present in the typical class 1 integron, was detected in twenty-two isolates (38.5%) among 57 class 1 integron positive. The variable regions of class 2 integrons in the two isolates presented the same gene cassette array: *dfrA1*-*sat1*-*aadA1*. For the VR of class 1 integrons, seven arrangements of gene cassettes were found containing different alleles of *dfrA* (resistance in the trimethoprim), *aadA* (resistance in the streptomycin), and *cmlA* (resistance to chloramphenicol). These arrangements were as follows (number of isolates): *dfrA1-aadA1* (10), *dfrA12-orfF-aadA2* (3), *dfrA5* (1), *dfrA17-aadA5* (2), *aadA1* (1), *dfrA1* (1), and *dfrA12*-*orfF*-*aadA2-cmlA1-aadA1-qacH-*IS*440-sul3* (2) (Figure 1).

## 4. Discussion

Among the 83 multiantimicrobial-resistant isolates, tetracycline resistance was observed in 78 isolates (93.9%). Indeed, a high rate of tetracycline resistance is a general trait of avian *E. coli* in Tunisia [19]. Genes of *tet* type were detected in 65 isolates. *tetA*, *tetB*, and *tetC* genes were observed in 59 (75.6%), 14 (17.9%), and one (1.2%) tetracycline-resistant isolates, respectively. Absence of these genes in some of our resistant isolates might be due to the occurrence of nontested or unknown resistance genes. The predominance of *tetA* and *tetB* genes was also reported by other studies while the *tetC* gene is rarely reported [20–22].

Among 67 sulfonamide-resistant isolates, 44 isolates contained genes of *sul* type; resistance in the remaining isolates might be due to chromosomal mutation in dihydropteroatic synthetase (DHPS) [23]. *sul1*, *sul2*, and *sul3* genes were detected in 31 (46.2%), 16 (23.8%), and 6 (8.9%) sulfonamide-resistant isolates, respectively. In agreement with our results, the gene *sul1* remains so far the most common gene followed by *sul2* gene; however, *sul3* is generally less frequent [24]. In contrast, in our previous study [25], the *sul3* gene was the most prevalent one in avian *E. coli* isolates. A single ESBL-producing isolate was identified among our collection. Recently, worldwide, ESBL-producing *E. coli* isolates have been reported in livestock especially from poultry origin [26]. In Tunisia, the presence of ESBL-producing *Enterobacteriales* has been previously reported in *E. coli* from poultry, pets, dromedary, and meat of various animals [9–12]. In those studies, ESBL production was detected by using selective protocols, while Soufi et al. [19, 25] have not identified any ESBL-producing *E. coli* isolate from a collection of 164 isolates. Therefore, since our *E. coli* isolates were recovered and randomly selected from a nonselective medium (such as MacConkey agar+cefotaxime [10, 11]), we were unable to identify high number of ESBL-producing isolates. The gene encoding the ESBL enzyme was identified as *bla*_CTX-M-1_, which is the most frequently reported ESBL gene in *E. coli* of animal origin in Tunisia and worldwide [27–29].

The role of plasmids in the dissemination of genes encoding antibiotic resistance is well documented. In addition, it appears that some plasmid types have a wide dissemination of power and are able to replicate in a wide host range [30]. Indeed, some plasmid types have been linked to the dissemination of genes encoding ESBL or fluoroquinolone resistance [30]. In our study, 29 (34.9%) strains harbored at least one plasmid. Plasmids belonging to the incompatibility groups, IncFIB, IncF, IncK, and IncI1-I*γ*, were the most prevalent; moreover, it is worth noting the presence of multiple replicons in 22 (26.5%) strains. These findings have been reported by other authors [30, 31]. Thus, the presence of multiple plasmids *per* strain represents an important genetic pool that might be used as a vector for antimicrobial resistance and/or virulence spreading within commensal intestinal *E. coli* isolates or other pathogenic genera such as *Salmonella* spp. [7, 27, 30–32]. Further genetic studies (hybridization or conjugation experiments) are needed to better understand the role of these plasmids. The remaining antimicrobial-resistant strains that did not harbor any of the investigated plasmid replicons likely harbored other nonsearched or novel replicons. Indeed, at least 27 plasmid incompatibility groups are recognized in *Enterobacteriaceae* [33]; however, we investigated only the 18 most common plasmid incompatibility groups. In addition, the used PCR-based replicon typing (PBRT) scheme targeting the replicons of the major plasmid families occurring in *Enterobacteriaceae* has several limitations and can fail to identify divergent or novel replicons [33]. It is also plausible that all those strains or at least part of them are plasmid free and that their genes encoding antimicrobial resistance are chromosomally located.

Integrons of class 1 were found in 57 isolates while class 2 only in two isolates; our results were in agreement with other works which showed the dominance of class 1 integron in *E. coli* isolates of animal and human origins [19, 25]. Class 1 integron is a dynamic genetic system encoding a functional integrase protein enabling integration and expression of several gene cassettes that are nonreplicative mobile elements, in the bacterial genome [5, 13, 14]. This genetic trait explains in part the high dominance of class 1 integrons in *Enterobacteriaceae*, particularly in resistant isolates and in a rich antimicrobial environment [13, 14, 19]. Classic class 1 integrons are mainly characterized by a 3′ conserved region containing *qacEΔ* (encoding resistance to quaternary ammonium) and *sul1* (encoding resistance to sulfonamides) genes; however, in our isolates only 22 (38.5%) out of the 57 class 1 integron-positive isolates harbored this sequence. This finding is in agreement with other studies reporting the absence of this region in class 1 integrons [19, 25, 32]. In a genetic point of view, it is plausible that crucial genetic rearrangements in class 1 integron happened. Indeed, this region was replaced by a ‘transposon-like' structure, a *qacH*-IS*440*-*sul3*, which could facilitate the dissemination of class 1 integron by a mechanism of transposition [34]. Owing to many financial limitations, we sequenced the variable regions of only 21 strains harboring the class 1 and 2 integrons; among them, one contained both types of integrons. In 20 class 1-positive isolates, the VR showed the presence of seven arrangements of gene cassettes. We found that the gene cassette array *dfrA1*-*aadA1* was the most frequently detected. Furthermore, two isolates harbored a long gene cassette array with unclassic 3′CS: *dfrA12*-*orf*F-*aadA2*-*cmlA1*-*aadA1*-*qacH*-*IS440*-*sul3*. This structure, known as *sul3*-associated integron type I, was also reported by other authors [25, 35]. This phenomenon of substitution of *qacEΔ*-*sul1* by *qacH*-*IS440*-*sul3* might be for perfection rather than change of function. Indeed, the inserted *qacH* and *sul3* genes code for the same functions as *qacEΔ1* (resistance the quaternary ammonium) and *sul1* (resistance to sulfonamides) genes, respectively. Genetically, this could be explained by the continuous use of the ammonium-quaternary and sulfonamides until current days in avian industries in Tunisia and worldwide [36]. A unique gene cassette array *dfrA1-sat1-aadA1* was identified in the variable region of class 2 integrons of two isolates. This structure is common in all class 2 integrons identified worldwide [32].

## 5. Conclusion

Taken together, these findings highlight the importance of intestinal avian *E. coli* as a reservoir of antibiotic resistance that is certainly linked to the excessive use of antibiotic in avian husbandry in Tunisia. This dramatical situation is not specific to Tunisia. Therefore, this is worrisome for global human health, especially with the increasing consumption of poultry meat in Tunisia and in other parts of the world owing to its relatively lower cost comparing to red meat.

## Figures and Tables

**Table 1:** Percentages of resistant isolates among the 83 *E. coli* to the tested antimicrobial agents in relation with the presence or absence of integrons.

Antimicrobial agents (*n* of resistant isolates)	Resistance frequencies (%)	Integron positive (58 isolates)	Integron negative (25 isolates)
		*N* (%)	*N* (%)
Streptomycin (62)	74.6	45 (72.5)	17 (27.4)
Kanamycin (7)	8.4	6 (85.7)	1 (14.2)
Gentamicin (0)	0	na	na
Tetracycline (78)	93.9	59 (75.6)	19 (24.3)
Trim/Sulfamethoxazole (58)	69.8	47 (81)	11 (18.9)
Sulfonamides (67)	80.7	53 (79.1)	14 (20.9)
Amoxicillin (55)	66.2	42 (76.3)	13 (23.6)
Amoxicillin/clavulanic acid (36)	43.3	25 (69.4)	11 (30.5)
Ticarcillin (27)	32.5	18 (66.6)	9 (33.3)
Cefotaxime (3)	3.6	2 (66.6)	1 (33.3)
Ceftazidime (2)	2.4	1 (50)	1 (50)
Imipenem (0)	0	na	na
Nalidixic Acid (46)	55.4	36 (78.2)	10 (21.7)
Ciprofloxacin (27)	32.5	22 (81.4)	5 (18.5)
Chloramphenicol (0)	0	na	na

na: not applicable.

**Table 2:** Resistance to different antimicrobial families according to the presence or absence of integrons.

Types of isolates	Number of isolates resistant to the following number of antimicrobial families:			
	<2^a^	3	4	>5^b^
Integron positive (*n* = 58)	2	10	16	30
Integron negative (*n* = 25)	12	4	3	6

^a^Integron-negative isolates were more frequently resistant to 2 or less families of antimicrobials than integron-positive isolates (*p* < 0.01). ^b^Integron-positive isolates were more frequently resistant to 5 or more families of antimicrobials than integron-negative isolates (*p* < 0.001).

**Table 3:** Resistance genes detected by PCR assays in integron-positive and integron-negative *E. coli* isolates.

Antimicrobial agent (number of resistant isolates)	Genes detected in isolates (number of isolates):	
	Integron positive (*n*)	Integron negative (*n*)
Sulfonamides (67)	*sul1* (18)	*sul1*(5)
	*sul2* (9)	*sul2* (1)
	*sul3* (1)	*sul3* (1)
	*sul1*+*sul2* (4)	
	*sul1*+*sul3* (3)	
	*sul2*+*sul3* (1)	
	*sul1*+*sul2*+*sul3* (1)	

Tetracycline (78)	*tetA* (34)	*tetA* (19)
	*tetB* (6)	*tetB* (2)
	*tetC* (1)	*tetA*+*tetB*(1)
	*tetA*+*tetB*(5)	

## Data Availability

The statistical data used to support the findings of this study are available from the corresponding author upon request.
